# Development and characterization of 26 novel microsatellite loci for the trochid gastropod *Gibbula divaricata* (Linnaeus, 1758), using Illumina *MiSeq* next generation sequencing technology

**DOI:** 10.7717/peerj.1789

**Published:** 2016-03-24

**Authors:** Violeta López-Márquez, Ricardo García-Jiménez, José Templado, Annie Machordom

**Affiliations:** 1 Biodiversidad y Biología Evolutiva, Museo Nacional de Ciencias Naturales (MNCN-CSIC), Madrid, Spain; 2 Molecular Systematics Lab, Museo Nacional de Ciencias Naturales (MNCN-CSIC), Madrid, Spain

**Keywords:** Microsatellite, Genetic structure, Population genetics, Cross amplification, Hybridization, *Gibbula varia*, *Gibbula rarilineata*, Mediterranean sea

## Abstract

In the present study we used the high-throughput sequencing technology Illumina MiSeq to develop 26 polymorphic microsatellite loci for the marine snail *Gibbula divaricata*. Four to 32 alleles were detected per locus across 30 samples analyzed. Observed and expected heterozygosities ranged from 0.130 to 0.933 and from 0.294 to 0.956, respectively. No significant linkage disequilibrium existed. Seven loci deviated from Hardy-Weinberg equilibrium that could not totally be explained by the presence of null alleles. Sympatric distribution with other species of the genus *Gibbula*, as *G. rarilineata* and *G. varia*, lead us to test the cross utility of the developed markers in these two species, which could be useful to test common biogeographic patterns or potential hybridization phenomena, since morphological intermediate specimens were found.

## Introduction

Top shells of the closely related genera *Gibbula* and *Phorcus* include common shallow-water and intertidal species found along rocky European coasts that possess limited dispersal capacity related to their encapsulated lecithotrophic development (Hickman, 1992). The diverse genus *Gibbula* seems to be para- or polyphyletic (Williams et al., 2010; Barco et al., 2013) and hence, poses problems for the identification and delineation of some constituent species. One of the most common Mediterranean species of this genus is *G. divaricata*, which is distributed throughout the Mediterranean (Templado, 2011) and Black seas (Anistratenko, 2005) in shallow, sheltered rocky bottoms, including some coastal lagoons. This species has a patchy distribution, with dense populations present in more favorable habitats, while absent in vast stretches of sandy beaches and exposed rocky coastlines. Morphologically, *G. divaricata* is similar to its congener *G. rarilineata*. The shells of both species have the same coloration pattern and are distinguished primarily by whorl convexity, being more pronounced in *G. divaricata*, while flatter with a concave base in *G. rarilineata* (Templado, 2011). Furthermore, these two species are known to form mixed populations in the same habitat, and morphologically intermediate forms can be found. However, according to Barco et al. (2013), *G. divaricata* is genetically more closely related to *G. varia* than to *G. rarilineata.*

These traits make *G. divaricata* an excellent species to study genetic differentiation at various geographic scales, connectivity among isolated populations and potential hybridization phenomena, using microsatellites as informative molecular markers. Furthermore, cross-amplification of *G. divaricata* microsatellites in *G. varia* and *G. rarilineata* may provide a useful tool for testing common biogeographical patterns or potential hybridization phenomena under a changing scenario of biotic homogenization of the Mediterranean Sea (Bianchi et al., 2013). To date, microsatellite marker development has only been implemented in one other species of this genus, *G. cineraria* (McInerney et al., 2011).

Understanding population connectivity in many marine species requires knowing the origin and trajectories of larvae among subpopulations, which is crucial for management and conservation (Pineda, Hare & Sponaugle, 2007; Cowen & Sponaugle, 2009). The encapsulated larval development of *G. divaricata* is fast: larvae leave the egg capsules 12 h post fertilization and live in the plankton for a very short period of time (Chukhchin, 1960). Thus, this supposedly restricted dispersal capacity suggests *G. divaricata* distribution may be a good predictor of barriers, allowing inferences of historical factors leading to current biogeographic and genetic patterns.

## Materials and Methods

A total of 30 specimens of *G. divaricata* were collected during 2013 in Saplaya Port (Valencia, Spain) (39°30′38.80″N, 0°19′5.11″W). The samples were collected by hand from the shallow rocky bottom. All specimens were preserved in absolute ethanol and stored at 4 °C until processed molecularly. The shell was cracked by applying pressure with a small hammer to allow ethanol to penetrate (which is sometimes prevented by the operculum) and fix the tissues within. Genomic DNA (gDNA) was extracted from approximately 2 mg of foot tissue close to the operculum. DNA was purified using the QIAGEN BioSprint 15 DNA Blood Kit (Qiagen Iberia S.L., Madrid, Spain), according to manufacturer’s protocol, including an RNase treatment. DNA was quantified using the Quant-iT dsDNA HS Assay read in the Qubit 2.0 fluorometer according to manufacturer’s instructions, and DNA aliquots at 2 ng/µl were made for subsequent genotyping analysis. DNA quality was checked on a 1% agarose gel. All samples were identified to the species level by morphological and molecular determination. Morphological characterization was based on shell features. Given the difficulty of morphological discrimination of some *G. rarilineata* specimens, molecular identification was additionally made by DNA barcoding, as suggested in Barco et al. (2013). A 658 base pair (bp) fragment at the 5′ end of the cytochrome c oxidase subunit I (COI) gene was amplified using the primer pair LCO1491 (Folmer et al., 1994) and COI-H (Machordom et al., 2003). Sequences, including those obtained from GenBank, were compared using the BLAST algorithm (Altschul et al., 1997).

For Illumina *MiSeq* next-generation sequencing, gDNA was extracted from the foot tissue of a single *G. divaricata* specimen, as described above. Briefly, isolated gDNA (50–100 ng) was singly digested with the restriction enzymes *AluI*, *RsaI* and *Hpy166II* (New England Biolabs, USA). After heat inactivation of the restriction enzymes, equal amounts of the three digests were combined in a single tube and the blunt ends were adenylated (+A) with Klenow (exo-) and dATP. After heat inactivation of the Klenow (exo-), the reactions were supplemented with ATP to a final concentration of 1 mM, and Illumina Y-adaptors were ligated to the ends with T4 DNA ligase. Digested, ligated DNA fragments were enriched for microsatellites by hybridizing to 3′-biotinylated oligonucleotide repeat probes (GT)_8_, (TC)_9.5_, (TTC)_7_, (GTA)_8.33_, (GTG)_4.67_, (TCC)_5_, (GTT)_6.33_, (TTTC)_6_, (GATA)_7_, (TTAC)_6.75_, (GATG)_4.25_, (TTTG)_5.25_, (TGTC)_4.5_, (TGTA)_6_, (TTTTG)_4.2_ and (TTTTC)_4.6_ (Integrated DNA Technologies) at 56 °C. Using streptavidin-coated magnetic beads, enriched fragments were isolated then amplified with *One Taq* polymerase and a pair of Illumina primers (one universal, one index). PCR products were analyzed on a 1.5% agarose gel and quantified with a Qubit 2.0 fluorometer. Equal amounts of each library were pooled and 300–600 bp fragments were recovered with Ampure beads (www.beckmancoulter.com). A library of these microsatellite-enriched fragments was prepared and sequenced by the Sequencing and Genotyping Facility at the Cornell Life Sciences Core Laboratory Center (CLC) for 2 × 250 paired end sequencing. Raw data was assembled using SeqMan NGen (v.11, Lasergene Genomics Suite; DNASTAR, Madison, Wisconsin, USA).

Selection of microsatellite-containing contigs generated from the Illumina *MiSeq* genomic dataset for primer design was done using the bioinformatics pipeline QDD version 3.1 (Meglécz et al., 2014). A total of 7.4 MB of data and 19,641 reads were obtained. After adapter sequences were trimmed, sequence similarities were compared by an ‘all against all’ BLAST (Altschul et al., 1997), implemented in QDD version 3.1, in which microsatellite motifs were soft masked. Sequences longer than 100 bp and containing perfect microsatellite motifs of at least five repetitions for any 2–6 bp motif were selected for further analyses. Nevertheless, some of the developed tetranucleotides (Gd-L1, Gd-L5, Gd-L16 and Gd-L22) behaved as dinucleotides, and four of the selected loci (Gd-L28, Gd-L30, Gd-L37 and Gd-L39) had microvariants. A total of 12,238 microsatellite-containing sequences were identified as potential markers. QDD then selected 2,892 sequences containing enough flanking sequence for primer design. Within these sequences, the most common repeat motifs were di- (35.76%), followed by tri- (33.48%), tetra- (29.06%), penta- (1.19%) and hexanucleotides (0.51%). Primers were designed using PRIMER3 v.0.4.0 (Untergrasser et al., 2012), with the following criteria: GC content 40–60%, product size 90–320 bp, primer length 18–27 bp and melting temperature between 59 and 61 °C with a maximum 3 °C difference between primer pairs. Primers could be designed for all 2,892 sequences. However, after checking for contamination against the NCBI nucleotide database using BLAST and comparing to known transposable elements using the online version of RepeatMasker v4.0 (Smit, Hubley & Green, 2013–2015), a total of 44 primer pairs were selected and synthesized as potential microsatellite markers. For primary screening, primers were first validated by successful PCR amplification and genotyping of 24 individuals from 11 different populations of *G. divaricata*, one individual for *G. varia* and 12 for *G. rarilineata*, using the same conditions for all three species.

A nested PCR amplification protocol successfully applied in other species, including the bivalve mollusc *Panopea abbreviata* (Ahanchédé et al., 2013) and the nemertean *Malacobdella arrokeana* (Alfaya et al., 2014), was used. Based on the expected sizes of amplification products, the scorability of electropherogram patterns and polymorphisms, 26 polymorphic microsatellite loci were chosen for further characterization.

To assess the polymorphism and population genetic parameters at these 26 loci, we genotyped 30 *G. divaricata* individuals collected at Saplaya Port. Nested PCRs were carried out in a total volume of 10 µl with 1×PCR Biotools Standard Reaction Buffer including 2 mM MgCl_2_, 0.5 µM forward and reverse primers, 0.2 mM of each dNTP, 1.5U DNA polymerase (Biotools), and 2 ng of template DNA. The primers included a 5′-end tag to facilitate the second amplification (in the forward primers) and to avoid stutters (in the reverse primers) (see Acevedo et al., 2009). PCR amplifications were performed in a Veriti™ Thermal Cycler (Applied Biosystems) under the following conditions: an initial denaturing step of 94 °C for 3 min, followed by 35 cycles of denaturation at 94 °C for 45 s, annealing between 50 and 60 °C (see Table 1) for 45 s and extension at 72 °C for 30 s, and a final extension of 10 min at 72 °C. PCR products were fluorescently labeled in a second round of PCR using the above amplification conditions. The forward primer in this reaction was 5′-TGACGACCCCATGCTACG-3′ (Acevedo et al., 2009) fluorescently 5′ end labeled with 6-FAM, NED, VIC or PET (see Table 1).

**Table 1:** *Gibbula divaricata* microsatellite characterization. Forward primers were 5′ end-tailed with 5′-TGACGACCCCATGCTACG-3′ and reverse primers were pig-tailed to facilitate genotyping with 5′-GTTTCTT-3′.

Locus name	Primer sequences (5′-3′)	Annealing temperature	Repeat motif	Alleles range (bp)	Na	Ho	He	*P*-value HWE	Accession number
Gd-L1	F 6-FAM-CTGACACTTGTTTAGCGACTCCT	56 °C	(TACA)_14_	132–264	19	0.586	0.747	0.149	KT880040
	R GAAAGGGAGATTACTCAAATATTCTG								
Gd-L3	F PET-TGGTAAAGTGCATTTTGATGTCTG	50 °C	(ACAG)_11_	132–160	7	0.519	0.747	0.559	KT880041
	R TCATGCAAATAATCATTC								
Gd-L5	F NED-GGAATTCCCATCACTCCAGA	60 °C	(ACAT)_12_	192–322	29	0.346	0.956	0.000^*^	KT880042
	R TCAAGTGATAAATTACTATGCCACG								
Gd-L6	F VIC-GACCCTCTTCTGACTACAGAACG	56 °C	(GACA)_7_	119–148	8	0.367	0.839	0.000^*^	KT880043
	R TGATTGACGCTATCACTTGACC								
Gd-L7	F VIC-TGTCATTCCAACTTCTAAAATGC	56 °C	(ACAG)_8_	150–174	7	0.467	0.769	0.099	KT880044
	R TGCAGTTTTAAATGACTCACCA								
Gd-L10	F 6-FAM-TGTGAAGCAGATATAGAGGCAATG	50 °C	(CAGA)_8_	134–178	9	0.357	0.693	0.544	KT880045
	R CCAGAAACAACTCTGAAACCA								
Gd-L11	F VIC-TTGAGAGGGAAACTATTGTAGGGT	50 °C	(CAGA)_7_	188–208	4	0.333	0.294	0.977	KT880046
	R AACCTCAAGAAGATGGCTCACT								
Gd-L15	F VIC-TGACTCGATTTCGTCGCTTT	56 °C	(GAGT)_9_	105–137	7	0.467	0.730	0.368	KT880047
	R TGAAATACATGCTAAGTCTAAGCCG								
Gd-L16	F NED-ACGAGTTCATATCATGAGAAAGTCA	56 °C	(ATCT)_15_	172–336	32	0.900	0.956	0.794	KT880048
	R CTTTTGCACGTGAGTTTATTGG								
Gd-L17	F 6-FAM-ACTGATGCCATTCTCAAGCA	56 °C	(TATC)_12_	164–272	14	0.176	0.915	0.000^*^	KT880049
	R TTGCAACTCACTACCTATTTATTCTGA								
Gd-L20	F 6-FAM-CAATGTTACGATGGACGGAA	56 °C	(TCCA)_8_	162–214	8	0.867	0.760	0.372	KT880050
	R AACAAGCATTTAGGCGCAAG								
Gd-L22	F VIC-GAGTCCGGGTATCCGAGG	56 °C	(TACA)_16_	196–280	20	0.633	0.923	0.039	KT880051
	R GATTGTACAGTCGCCTGTGGT								
Gd-L23	F 6-FAM-TTGCCACAGAATGCAAACTAA	56 °C	(TAAG)_7_	153–181	8	0.700	0.800	0.693	KT880052
	R GACCTCATGACTACTGTGAACTTACTC								
Gd-L26	F 6-FAM-AAATTCTGATGACACATCGTTT	56 °C	(GACA)_7_	151–223	11	0.286	0.846	0.000^*^	KT880053
	R ACTCCCGTCTTATGGGCCT								
Gd-L28	F NED-AGTTTGTTCCTTTCCTCCACAG	56 °C	(GAT)_15_	138–190	18	0.724	0.927	0.029	KT880054
	R CCTTCAACGTTTGATAAGTTCG								
Gd-L29	F NED-AGTCTCTTGGTGCAGGGAAT	56 °C	(ATG)_8_	186–252	13	0.458	0.875	0.003	KT880055
	R TGTCGCAAACAACATCAACG								
Gd-L30	F PET-ATGCACATTGTTTTAGACGGC	56 °C	(AGA)_9_	150–189	11	0.933	0.874	0.785	KT880056
	R ACTATACGTTGTACCCAATCGAC								
Gd-L32	F PET-GGATACATTTATCAAACACCCACT	56 °C	(GAA)_2_ (GAT)_2_	158–182	9	0.633	0.682	0.681	KT880057
	R GCTTGCAATCTTCACCAACTC		(GAA)_11_						
Gd-L34	F NED-TTATTTATTGCCTTTGCGTAGC	56 °C	(CAA)_8_	122–134	5	0.182	0.447	0.000^*^	KT880058
	R CGTGTTATTGGCTTCCTCCA								
Gd-L37	F 6-FAM-TTTATAAGAAAATGTGGGCAGCA	56 °C	(GAA)_11_	226–249	12	0.800	0.853	0.814	KT880059
	R CACAACCGACACGAAACTTG								
Gd-L38	F 6-FAM-GCTTAAGTGTCCAGATAACATTCTACC	56 °C	(AAC)_14_	138–192	17	0.733	0.888	0.082	KT880060
	R CGATCGAAGTTTTCTAGGTCATACATT								
Gd-L39	F NED-ACGCCGCTACAGCATAAAAC	50 °C	(AAC)_10_	305–335	11	0.130	0.789	0.000^*^	KT880061
	R TGCTGGTATGATGAAATCTGTC								
Gd-L40	F 6-FAM-TTCTTTATTTTGATGTTGCAAAACTT	56 °C	(CTA)_9_	169–220	13	0.837	0.853	0.979	KT880062
	R CGTATCCTTGTTCAAGGTCTCT								
Gd-L41	F NED-ACGATACAACCACCTGAGCA	56 °C	(AGA)_11_	109–133	9	0.862	0.724	0.937	KT880063
	R TCGAAATTAGATAAATACCATGTTTCA								
Gd-L42	F PET-GCGAAGTTTCGGTATGAGAATC	56 °C	(AGA)_8_	113–173	9	0.310	0.618	0.000^*^	KT880064
	R TGGCGATAAAATACATAACATGA								
Gd-L43	F NED-CTACCTTGATACTGATCGGTGGAG	56 °C	(ATG)_9_	189–222	12	0.862	0.847	0.945	KT880065
	R TTATCGAGAGTACAAGTCAGTGATAGA								

**Notes.**

bp base pairs F Forward R Reverse Na number of alleles Ho observed heterozygosity He expected heterozygosity HWE Hardy-Weinberg equilibrium

* Loci deviating from HWE equilibrium after null allele and sequential Bonferroni corrections.

Fluorescently labeled PCR products were run on an ABI PRISM 3730 DNA Sequencer (Applied Biosystems), scored using the GeneScan-500 (LIZ) size standard and analyzed with the GeneMapper software (Applied Biosystems).

The sequences of the 26 selected loci were deposited in GenBank (Accession numbers in Table 1). Number of alleles (Na), observed (Ho) and expected (He) heterozygosities, test of Hardy-Weinberg equilibrium (HWE) and linkage disequilibrium were calculated with GENEPOP 4.0 (Rousset, 2008) and GenAlEx (Peakall & Smouse, 2006; Peakall & Smouse, 2012) softwares. If necessary, significance values were adjusted for multiple comparisons using sequential Bonferroni corrections (Rice, 1989). Data were reviewed for null alleles and scoring errors using MICROCHECKER (Van Oosterhout et al., 2004). The probabilities of exclusion for each locus and for increasing combinations of the 26 loci were also tested using GenAlEx.

## Results

The repeat motifs of 25 of the 26 developed loci exactly matched the probes used, indicating high enrichment efficacy. A total of 322 alleles (4–32 alleles per locus) and a mean number of 12.4 alleles per locus were detected across the 26 loci in the 30 genotyped individuals confirmed to be *G. divaricata* by COI analysis. Observed and expected heterozygosites ranged from 0.130 to 0.933 and 0.294 to 0.956, respectively (Table 1). No significant pairwise linkage disequilibrium was observed among the loci (*p* ≥ 0.013; 0.05 ≥ *α* ≥ 0.00017 following Bonferroni sequential correction). Seventeen loci were in agreement with Hardy-Weinberg Equilibrium (HWE) while nine significantly deviated from HWE (0.979 ≥ *p* ≥ 0.005; 0.05 ≥ *α* ≥ 0.0029 following Bonferroni sequential correction). A possible explanation for Hardy-Weinberg disequilibrium could be the existence of null alleles. To test this hypothesis, null allele frequencies were first evaluated by MICROCHECKER resulting in relatively high values (reaching 30%) as occurs in other marine invertebrates (Hare, Karl & Avise, 1996; Hedgecock et al., 2004). Then a new analysis of HWE equilibrium was performed including null alleles. Two (Gd-L28 and Gd-L29) of the nine loci were now in equilibrium, while the rest (Gd-L5, Gd-L6, Gd-L17, Gd-L26, Gd-L34, Gd-L39 and Gd-L42) still were not. A high number of alleles (from 11 to 29) was detected in four of these loci, which may explain the departure from HWE, as the number of samples analyzed (max. 30) may be insufficient to reveal all possible genotypic combinations. Also, the number of null alleles may be due to PCR failures caused by unstable flanking sequences (e.g., from mutations at PCR primer binding sites), thus leading to an underestimation of allele frequencies and heterozygosity (McInerney et al., 2011). In any case, once more populations are analyzed, the loci in disagreement with HWE will have to be tested for signs of selection pressures.

The loci diversity found gave rise to a high potential for analyses of kin relationships. The probability of excluding two specimens as related when they are not was 59% with the first locus (Gd-L1) and increased to 98% with the addition of the two following loci, Gd-L3 and Gd-L5. A 100% probability of exclusion was attained once nine of the 26 loci were included in the analysis.

Cross-amplification analysis, under the same PCR parameters used to amplify *G. divaricata*, showed that four of 26 loci, Gd-L1, Gd-L7, Gd-L39 and Gd-L43, successfully amplified fragments from the Spanish specimen of *G. varia*. Twelve *G. rarilineata* individuals collected from Mediterranean (Spain, Malta and Greece) and Black Sea (Bulgaria, Romania, Georgia and Ukraine) populations were successfully amplified and genotyped for six loci (Gd-L1, Gd-L10, Gd-L15, Gd-L23, Gd-L34 and Gd-L43). For two other loci, Gd-L11 and Gd-L22, three and four individuals, respectively, were also successfully amplified. Although the Gd-L7 primers produced products in *G. rarilineata*, no interpretable genotypes were found.

## Discussion

Microsatellites have been widely used in different organisms, greatly supporting population genetic studies. Their development has improved in recent years, achieving greater efficiency at lower costs. However, their isolation remains problematic in certain taxa, such as in molluscs, a finding that has never been fully investigated or explained (McInerney et al., 2011). In this study, using the NGS Illumina *MiSeq* platform, we have successfully developed 26 loci that all work well in the targeted species (*G. divaricata*), with some loci able to cross amplify in closely related species (*G. varia* and *G. rarilineata*).

Overall, the number and polymorphism of the *G. divaricata* microsatellite markers characterized here will aid future studies of population genetics and structure, gene flow, kinship relationships and demography for this species. Although this species is widely distributed throughout the Mediterranean and Black seas (Templado, 2011; Anistratenko, 2005), it is thought to have limited dispersal capacity. Therefore, considering the isolation between these two basins and the different barriers described for the Mediterranean, suggesting a number of distinct biogeographic sectors or eco-regions within it (Bianchi, 2007; Giakoumi et al., 2013), differentiation among *G. divaricata* populations with a marked genetic structure can be hypothesized. Hence, the markers developed here will be useful for testing these hypotheses and for assessing gene flow and connectivity among populations. Moreover, the high theoretical probability of exclusion allows for more accurate studies about larval retention and recruitment that, coupled with previous data about connectivity, may provide more insightful data for better management and conservation efforts.

Furthermore, given the preliminary success of cross-species amplifications in *G. varia* and *G. rarilineata*, modifications of the tested PCR conditions (mainly annealing temperature and DNA and MgCl_2_ concentrations) could result in additional useful markers that may help elucidate the relationships among species of this genus or test potential hybridization phenomena among closely related species. The presence of morphologically intermediate specimens suggests relaxation of reproductive barriers or secondary contacts between the currently sympatric species *G. divaricata* and *G. rarilineata* or *G. divaricata* and *G. varia*. Here we have classified samples based on morphological characters and maternal lineages; however, these three species share some of the developed markers, thus the actual level of hybridization or introgression could be assessed more precisely in future studies.

## Additional Information and Declarations

### DNA Deposition

The following information was supplied regarding the deposition of DNA sequences: GenBank accession numbers KT880040to KT880065.
